# To Flip or Not to Flip: Learning Style Preferences among Millennial Physician Assistant Students

**DOI:** 10.7759/cureus.13467

**Published:** 2021-02-21

**Authors:** Katherine Schultz, Alicia Schaffer, Rebecca Rebman, Anthony Shanks

**Affiliations:** 1 Obstetrics and Gynecology, Indiana University School of Medicine, Indianapolis, USA; 2 Medicine, Indiana University School of Medicine, Indianapolis, USA

**Keywords:** styles, education, flipping, teaching, obstetrics

## Abstract

Introduction: Presenting material in a manner that is most palatable to students is important to improve the learning process. We evaluated the efficacy of different teaching styles including the flipped classroom and assessed the learning style preferences of a cohort of medical learners in a preclinical obstetrics and gynecology course.

Methods: We conducted three teaching sessions with 35 physician assistant students. A different teaching style was implemented for each session including a traditional lecture with interactive learning technology augmentation, a flipped classroom, and a hybrid approach incorporating lecture and group work. Students were surveyed using a Likert scale regarding the efficacy of the format, clinical relevance of the material, and their learning preference for future sessions.

Results: Students rated the traditional approach as the most effective, most relevant, and most preferred method. Students preferred the flipped classroom least, but they rated it as slightly more effective and relevant than the hybrid approach.

Conclusion: The teaching style of various coursework including the preclinical obstetrics and gynecology curriculum may not need to be altered for millennial learners. This study showed the flipped classroom was the least favored teaching style and that there was a marked preference by students for a more traditional didactic lecture.

## Introduction

Traditionally, preclinical medical education has been firmly based on the “sage on the stage” method in which an expert lectures a group of students, with or without educational aids (e.g., chalkboards, projectors, PowerPoint). In this style, the learning is passive, with student interaction typically limited to a brief question-and-answer period at the conclusion of the lecture. In the early 1990s, this educational paradigm began to shift, and educators were encouraged to function as facilitators that challenged their students to interact with, rather than simply memorize, new information [1].

As the majority of medical learners now belong to the Millennial generation, technology and novel learning techniques have become increasingly integrated into traditional teaching styles. This change is to improve both student performance and student satisfaction with their education. It has been proposed that Millennial learners prefer simulation, interactive group activities, and workshops as compared to traditional didactic modalities such as lectures or Socratic questioning [2]. Among these newer and increasingly utilized techniques are podcasts, interactive tools to augment traditional lecture formats, game-based learning, and the flipped classroom [2,3].

The flipped classroom, also called an inverted classroom, is a teaching method in which new material is first presented to students outside of the classroom setting in the form of pre-assigned work such as written materials or videos [2,3]. The same material is later formally reviewed during the class time via instructor-facilitated discussions and case-based scenarios [2,3]. The flipped classroom is designed to encourage active learning rather than passive learning which is typically seen in traditional didactic lectures. The use of the flipped classroom has become a trend in medical education in recent years. This has been promoted by the concept that the flipped classroom is a teaching format which encourages self-directed learning, application of concepts, and learner-centered instruction. However, the flipped classroom method of teaching has been noted to take more time for educators to design and maintain than traditional didactic lectures [4]. The debate is still occurring within education on whether the switch to flipped classroom is more beneficial for students and if the switch from traditional didactic lectures is worth the time and effort for faculty.

Previous studies have examined the effect of the flipped classroom teaching technique on student performance in both undergraduate and graduate medical education settings. The results in these studies have ranged from demonstrating equivalence between the flipped classroom and traditional lecture-based learning to showing a marked improvement in performance when using the flipped classroom format [4-12]. While not always a primary focus, several studies have also considered student satisfaction related to the flipped classroom. The results of these studies have also varied significantly from complete dissatisfaction to complete support of the flipped classroom as an educational modality [5-9,13-16]. Overall, there are currently contradictions and disagreements over the efficacy of the flipped classroom and student preference towards the flipped classroom in the literature.

Historically, there has not been an association between student preference of the teaching format and performance [17]. However, there is a positive correlation between student engagement with the educational process and academic performance [18]. Presenting material in a manner that is most palatable to students can improve engagement with the learning process. Furthermore, this increased engagement can result in improved test scores. Given that educational style can influence student engagement and, ultimately, performance, further examination of student satisfaction with this now-popular educational method is worthwhile. This study was therefore designed as an effort to clarify student satisfaction and evaluate students’ perceptions of effectiveness of the flipped classroom in comparison to an augmented traditional lecture-based teaching and a hybrid approach incorporating group work and lecture within a medical education curriculum.

## Materials and methods

In the academic year of 2016-2017, 35 physician assistant students were instructed in several topics over three separate classroom sessions. The students were in the process of learning the obstetrics didactic curriculum at Indiana University and didactics were in-person. Students were not previously exposed to the education topics prior to these sessions.

Each classroom session utilized a different teaching technique. A traditional didactic lecture was created using a case-based format for the topics of preterm birth, placenta previa, and preeclampsia. This didactic lecture was presented with Nearpod, an interactive presentation tool that can augment existing PowerPoint or PDF lectures. Created in 2011, Nearpod is a web-based presentation device that allows learners to view presentations on their laptop or smartphone. It has gained popularity in elementary schools given its ease in embedding videos and questions into traditional PowerPoint lectures. Lecture material was presented and Nearpod used to provide quizzes for retrieval practice during the lecture. The flipped classroom format was used to teach the topic of multiple gestation. This session employed educational material from the Association of Professors of Gynecology and Obstetrics (APGO). Students prepared for the session prior to class by reviewing assigned material. The students worked in small groups on clinical cases during regular class times. Lastly, a hybrid technique incorporating lecture and group work was applied to teach the topic of alloimmunization. All three educational sessions were provided by the same board-certified Obstetrics and Gynecology physician, and all 35 students attended all sessions. 

Following completion of the three sessions, a survey was administered to and completed by each member of the cohort. This survey used a 5-point Likert scale to assess the perceived effectiveness of each teaching method, the appropriateness of the material for level of training, the efficacy of group discussion in enhancing the material, and the satisfaction with each teaching style. A 5-point Likert scale was used to quantify each response. A score of 1 was used to designate “least effective,” while a score of 5 denoted “most effective.” Ordinal responses were then compared using the Friedman test. This study was approved by the Institutional Review Board at Indiana University.

## Results

Surveys assessing the efficacy of each teaching method were collected from 100% of the 35-member cohort. The mean student age was 26 years (range 22-42). The traditional case-based lecture using Nearpod was rated as the most effective teaching method (mean score 4.57; max 5). The hybrid technique that incorporated both lectures and group work was rated as the least effective (mean score 3.0; max 5). The flipped classroom format was rated as slightly more effective than the hybrid technique (mean score of 3.03; max 5). The difference in scores between teaching techniques was statistically significant (p < 0.01). The relevancy of material for level of training was also most highly rated with the traditional case-based style (mean score 4.69). This was compared to both the flipped classroom (mean score of 3.63) and the hybrid technique (mean score 3.06). Again, this difference in scores was statistically significant (p < 0.01). Students were also asked to rank each teaching style according to their preference for future lessons. A statistically significant majority of students (28 students/35; p < 0.01) preferred the traditional case-based lecture format using Nearpod. A smaller minority of students preferred the hybrid technique (four students/35). The flipped classroom was the least preferred method with only two students (out of 35) preferring this teaching style over the other formats.

## Discussion

This study was designed to assess students’ perceptions of the flipped classroom and add to the growing body of literature about this teaching technique. The results of our study demonstrated a preference for a more traditional educational format. Students rated the traditional approach as the most effective, most relevant, and most preferred method. The flipped classroom was the least favored teaching modality among the three included in this study. 

Several factors could have contributed to our results. First, a particularly engaging lecturer can create a more appealing and interactive experience to learn material in a didactic session as opposed to the completion of a web-based module or the reading of an article independently can create in a flipped classroom. Another factor to consider is that there is a learning curve for students to succeed in the flipped classroom. Students newly exposed to the flipped classroom may experience an adjustment period prior to showing increased performance on exams [12]. It is possible that if the flipped classroom was utilized for a longer period of time that students would become more efficient at the process and preference for it would increase. Lastly, the obstetrical topics chosen for the flipped classroom session may not have been ideal for the self-learning required in pre-assigned work. It’s possible that the topic chosen for the flipped classroom session was more suited for the traditional lecture style. 

A vital part of the flipped classroom is student preparation outside of class. In a previous study, students preferring the traditional lecture over the flipped classroom cited the reason for their preference being their dislike towards the additional time required outside of class for the flipped classroom session in order to complete the pre-assigned work [10]. It has been suggested that teachers limit pre-assigned work in flipped classroom sessions to 20 minutes [10]. We did not evaluate the time it took students to complete the pre-assigned work, but this could have influenced student preference for the traditional lecture. Additionally, it’s important to realize that there can be significant noncompliance with completing assigned work ahead of flipped classroom sessions [19]. This non-compliance can reduce participation, satisfaction, and performance in the group setting. Implementing a quiz at the beginning of the flipped classroom session has been shown to improve student noncompliance [10]. This quiz allows instructors to identify and address learning gaps that might make the session confusing and less productive. Our study did not assess student compliance with pre-assigned work and thus this effect could not be quantified. These factors may explain student preference for traditional lectures. 

This study has several strengths. First, all lectures and educational sessions were conducted by the same instructor. This reduces the potential that differences between lecturers could influence the results. Also, the flipped classroom modules in our study utilized materials that have previously been published by APGO. These materials were crafted by APGO to optimize student performance. The use of pre-designed and already studied materials reduces confounding that could be introduced by the use of new or individually assembled materials. Additionally, this study included several different educational formats with the same cohort of students over a relatively limited time period. This reduced the variance that could occur with different groups of students or with the same group over a longer period of time. We also were able to achieve full participation in all educational sessions from all participants. The 100% response rate to our survey means there was no loss to follow up. 

The study also does have limitations. As previously mentioned, there was no assessment of student compliance with completion of preparation materials prior to formal sessions. Not completing pre-assigned materials could have reduced student engagement and satisfaction with the flipped classroom approach. Similarly, student performance was not evaluated and thus the effect of the students’ preference for the more traditional format on performance cannot be assessed. Also, the traditional format utilized in this study was augmented with Nearpod. Nearpod was not used in most other studies as the “traditional” educational model. There could therefore be some variation in technique between our version of a traditional lecture from a more conventional “traditional” method. This factor may reduce the ability of this study to be compared against similar studies. In addition, different modalities were used for different topics. Our study does not account for any differences between the subject matter that could make one topic more innately complicated, abstract, or difficult to understand than another topic. Also, our cohort was comprised of physician assistant students. Therefore, our results may not be generalizable to other or graduate medical education cohorts. Finally, our sample size was small, which could result in lower generalizability or reproducibility of our findings. 

There are many future scholarly applications and research opportunities for this topic. This could include repeating our study among a different cohort of Millennial learners such as medical students or Obstetrics and Gynecology residents to assess for differences in lecture preference. Also, our study could be repeated with an objective evaluation of student performance in order to correlate student preference towards educational method with their performance. In addition, this study could be repeated with an assessment of student compliance with the completion of the out-of-class pre-assigned work prior to the flipped classroom session in order to examine the impact of completion of pre-assigned work with student preference for teaching technique. At the very least, utilizing the interactive format of the Nearpod presentation applications allows for increased engagement of the traditional lecture.

## Conclusions

This study showed that the flipped classroom was the least favored teaching style and that there was a marked preference by students for a more traditional didactic lecture using Nearpod. Based on student satisfaction, the teaching style of various coursework including the preclinical obstetrics and gynecology curriculum may not need to be altered for Millennial learners. Medical educators need to consider the efficacy of teaching style and student preference when preparing lectures in order to maximize learning potential. More research will need to be done to further clarify the mixed results of student preference in the literature.
